# Ameliorative Effect of Itaconic Acid/IRG1 Against Endoplasmic Reticulum Stress-Induced Necroptosis in Granulosa Cells via PERK-ATF4-AChE Pathway in Bovine

**DOI:** 10.3390/cells14060419

**Published:** 2025-03-12

**Authors:** Xiaorui Yang, Yue Chen, Xinzi Wang, Gaoqing Xu, Hongjie Wang, Xinqi Shu, He Ding, Xin Ma, Jing Guo, Jun Wang, Jing Zhao, Yi Fang, Hongyu Liu, Wenfa Lu

**Affiliations:** 1 Key Laboratory of Animal Production, Product Quality and Security, Ministry of Education, Jilin Agricultural University, Changchun 130118, China; yangxiaorui2020@163.com (X.Y.); cy463374734@163.com (Y.C.); wxz879978434@163.com (X.W.); xugaoqing1995@163.com (G.X.); 13287712312@163.com (H.W.); turbo24777@163.com (X.S.); dinghe1103@126.com (H.D.); maxin3202@163.com (X.M.); jgchn@163.com (J.G.); junwang2004@126.com (J.W.); jlndzjing@126.com (J.Z.); fangyi@iga.ac.cn (Y.F.); 2 Key Laboratory of Utilization and Protection of Beef Cattle Germplasm Resources, Jilin Agricultural University, Changchun 130118, China; 3 Jilin Province Engineering Laboratory for Ruminant Reproductive Biotechnology and Healthy Production, College of Animal Science and Technology, Jilin Agricultural University, Changchun 130118, China

**Keywords:** itaconic acid, ERS, granulosa cells, necroptosis

## Abstract

The necroptosis of granulosa cells has been proven to be one of the important triggers of follicular atresia, which is an important cause of reduced reproductive capacity in cows. The rapid growth of granulosa cells is accompanied by endoplasmic reticulum stress (ERS), leading to granulosa cell death. However, the link between ERS and necroptosis, as well as its mechanism in bovine granulosa cells is still unclear. Itaconic acid is an endogenous anti-inflammatory and antioxidant small-molecule compound that can alleviate ERS. Therefore, the aim of the current study is to evaluate the effect of ERS on necroptosis and investigate the ameliorative effect of itaconic acid against ERS-induced necroptosis in granulosa cells. Bovine granulosa cells were treated with tunicamycin (Tm) to induce ERS. After the addition of the necroptosis inhibitor Nec-1 and the detection of the necroptosis inducer acetylcholinesterase (AChE), flow cytometry, transmission electron microscopy, and mass spectrometry were used to analyze the expression of itaconic acid and IRG1 in the granulosa cells. In addition, the role of the PERK pathway downstream of ERS in ERS-induced necroptosis was also investigated. We report here that ERS can induce necroptosis in granulosa cells. Itaconic acid supplementation significantly attenuates the effect of ERS-induced damage. In summary, this research provides a scientific basis and a drug reference for treating follicular atresia and improving bovine reproductive capacity.

## 1. Introduction

Ovarian granulosa cells play a crucial role as the primary structural components of follicles, intricately involved in follicle development and oocyte maturation. Necroptosis, a phenomenon observed in human granulosa cells, myocytes, and auditory cells, is programmed cell death mediated by RIPK1, RIPK3, and MLKL [1]. In human granulosa cells, specific isoforms of acetylcholinesterase (AChE) can induce necroptosis [2,3]. Recent studies have demonstrated that the necroptosis of granulosa cells directly contributes to reproductive disorders, such as follicular atresia and premature ovarian failure, impacting the reproductive efficiency of cattle [4,5,6].

During follicular growth, increases in the rates of oocyte growth and granulosa cell proliferation may lead to hypoxia, which can cause endoplasmic reticulum stress (ERS), and ultimately result in granulosa cell death [7,8]. The endoplasmic reticulum is an organelle found in eukaryotic cells that is primarily responsible for the synthesis and folding of secreted proteins or membrane proteins, lipid metabolism, and calcium storage. However, different physiological and stress conditions can disrupt endoplasmic reticulum homeostasis, resulting in the accumulation of misfolded proteins in the endoplasmic reticulum, a condition known as ERS [9,10]. Due to the accumulation of unfolded or misfolded proteins, cells activate the unfolded protein response (UPR), which is initially protective, but can become detrimental if ERS is severe and long-lasting [11]. The PERK-ATF4 pathway is the most active in UPR and is closely related to ovarian granulosa cell death [12,13]. ERS has been identified as the principal instigator of necroptosis in granulosa cells. However, the molecular mechanism of ERS-induced necroptosis in bovine granulosa cells is still unclear [14,15,16,17].

The alleviation of ERS can effectively inhibit necroptosis and has been proven in liver, epithelial, and other cells [18,19,20]. However, the current mitigation methods are usually exogenous substances or targeted genes, which may cause metabolic disorders in animals. Therefore, we thought that finding a safe method to alleviate ERS-induced necroptosis in granulosa cells is indispensable. We hypothesized that an endogenous metabolite, itaconic acid, would be an efficient method to ameliorate the adverse effects of ERS-induced necroptosis in granulosa cells. Itaconic acid is produced through the enzymatic action of aconitate decarboxylase, alternatively recognized as immune-responsive gene 1 (IRG1). Previous studies have found that itaconic acid and its derivatives can alleviate inflammation, stress response, and cell death, serving as an important bridge between metabolism, inflammation, oxidative stress, and the immune response [21,22,23]. However, the protective effect of itaconic acid against ERS-induced necroptosis in bovine ovarian granulosa cells is still unclear.

In the current study, an ERS model was established using the inducer tunicamycin (Tm), and cell viability assays, flow cytometry, and transmission electron microscopy were used to verify that Tm induces the necroptosis of bovine granulosa cells through AChE. In addition, we evaluated the expression of the PERK-ATF4 pathway and IRG1 in itaconic acid at the transcriptional and translational levels using RT-qPCR and Western blot analysis, respectively. Finally, we used Ellman mass spectrometry to examine the expression of itaconic acid in order to reveal the molecular mechanism of its protective effect on the ERS-induced necroptosis of bovine granulosa cells and provide a new innovative method for improving animal reproductive efficiency.

## 2. Materials and Methods

### 2.1. Experimental Design and Ethics Statement

In order to prove that itaconic acid could protect granulosa cells against ERS-induced necroptosis, we used Tm, an ERS inducer, to establish an ERS model and performed four experiments. The first experiment was conducted to check the effect of Tm on bovine granulosa cells. In this experiment, four different concentrations of Tm (2.5, 5, 7.5, and 10 μg/mL) were used at different time periods (4, 8, 12, and 24 h). Based on the results of this experiment, 5 μg/mL Tm treatment for 8 h was chosen for further experiments.

The second experiment was conducted to study whether the necroptosis inhibitor Nec-1 and the AChE inhibitor HupA could protect against ERS-induced necroptosis by Tm in bovine granulosa cells. Four different concentrations of Nec-1 (20, 30, 40, and 50 μM) and HupA (5, 10, 15, and 20 μM) were used at different time periods (12 and 24 h). Based on the results of this experiment, 30 μM/mL Nec-1 treatment for 12 h and 20 μM HupA treatment for 24 h were chosen for further experiments.

In the third experiment, we checked whether itaconic acid can alleviate ERS-induced necroptosis. We detected the expression of itaconic acid and IRG1 in the bovine granulosa cells after treatment with Tm. We also investigated the effect of IRG1 knockdown using siIRG1-396 on ERS-induced necroptosis. We finally supplemented the itaconic acid derivative 4-OI at four different concentrations (50, 100, 150, and 200 μM), and cell viability was monitored. Based on the results of this experiment, 200 μM 4-OI treatment for 12 h was chosen for further experiments.

The fourth experiment was conducted to investigate whether the PERK-ATF4 pathway can participate in regulating ERS-induced necroptosis; both PERK and ATF4 knockdowns were performed, and the PERK-ATF4 pathway and its downstream signal IRG1 were evaluated at both the transcriptional and translation levels.

All the experiments in the current study were performed according to the guidelines of Jilin Agricultural University (Approval ID: 20230824001).

### 2.2. Primary Cell Culture

The ovaries of healthy cattle collected from a slaughterhouse were placed in 37 °C normal saline and sent to the laboratory within 4 h. The ovaries were washed with normal saline containing 1% penicillin/streptomycin (Sangon Biotech, Shanghai, China), and follicular fluid from 3–8 mm follicles were aspirated and centrifuged at 1000 rpm for 5 min. The cells were washed 2–3 times with PBS containing 1% penicillin/streptomycin and centrifuged again. The collected granulosa cells were cultured on a 90 mm plate containing DMEM/F-12 medium (Gibco, Waltham, MA, USA) supplemented with 10% FBS and incubated for 24 h at 37 °C under 5% CO_2_.

### 2.3. Cell Viability Assays

The granulosa cells were cultured in 96-well plates (2 × 10^4^ cells/well) and incubated at 37 °C under 5% CO_2_, and then treated either with the ERS inducer Tm (Sangon Biotech, Shanghai, China), the necroptosis inhibitor Nec-1 (MedChemExpress, Shanghai, China), the AChE inhibitor HupA (MedChemExpress, Shanghai, China), or itaconic acid derivative 4-Octyl itaconate (MedChemExpress, Shanghai, China) for 4, 8, 12, and 24 h. Then, CCK-8 (APEXBIO, Houston, TX, USA) solution and DMEM culture medium were added to each well at a ratio of 1:10 and incubated in the dark for 4 h. Absorbance was measured at 450 nm using a microplate reader (BioTek, Winooski, VT, USA).

### 2.4. Quantitative Reverse Transcription PCR (qRT-PCR)

Total RNA was fixed from pellet cells using Trizol reagent (Takara, Tokyo, Japan), and then reverse-transcribed to cDNA using PrimeScript™ RT kit (Takara, Tokyo, Japan). SYBR^®^ Premix Ex Taq™ II (Takara, Tokyo, Japan) was used for real-time fluorescence quantification. Relative gene expression was analyzed by the 2^−ΔΔCt^ comparison method. Primer sequences are provided in Table S1.

### 2.5. Western Blot Assay

The granulosa cell pellets were lysed using RIPA buffer (Beyotime, Shanghai, China), centrifuged at 4 °C for 15 min at 12,000 rpm, and the supernatant was collected. The protein concentration in the supernatant was determined by the BCA Protein Assay Kit (Beyotime, Shanghai, China) according to the manufacturer’s instructions. The protein samples were heated in 5× protein loading buffer for 8 min, and then the proteins were separated by 10–12% SDS-PAGE and transferred to nitrocellulose membranes (Merck Millipore, Darmstadt, Germany). After blocking with PBS-blocking buffer (LI-COR Biosciences, Lincoln, NE, USA) for 1.5 h, they were incubated overnight at 4 °C with primary antibody (Table S2). After washing with 5×-TBST, the secondary antibodies were incubated for 1 h. Band density was analyzed using a chemisope imaging system (CLiNX Science Instruments, Shanghai, China).

### 2.6. Flow Cytometry Assay

The treated granulosa cells were collected with trypsin, and the collected cells were washed 2–3 times with PBS. The washed cells were placed in 200 μL of 1× buffer, supplemented with 5 μL FITC Annexin V and 5 μL PI (BD Biosciences, San Jose, CA, USA) in the dark for staining, and incubated at 37 °C for 15 min. Then, 200 μL of 1× buffer was added, and the cells were screened for necroptosis using a flow cytometer (ACEA Biosciences, Hangzhou, China).

### 2.7. Transmission Electron Microscopy

The granulosa cells were suspended in an transmission electron microscope fixative (Servicebio, Shanghai, China). The cells were then pelleted and resuspended in 0.1 M PBS (pH 7.4) and washed three times for 3 min each time before being pelleted again. The pellets were picked up and suspended in 1% agarose solution (Bioteke, Beijing, China) in an EP tube before they was allowed to solidify. A resin block was sliced into 60–80 nm ultra-thin sections using an ultramicrotome (Leica, Shanghai, China), and then placed on 150-mesh copper grids coated with Formvar film. The sections were stained with 2% uranyl acetate saturated alcohol for 8 min in a dark room, and then washed three times with 70% alcohol and three times with distilled water, followed by staining with 2.6% lead citrate for another 8 min. After washing the sections 3 times with distilled water, they were dried overnight at room temperature. Finally, the images were analyzed using a transmission electron microscope.

### 2.8. Transient Cell Transfection

Transfection was performed when the granulosa cells had reached 70% fusion using siRNAs (Table S3) designed by GenePharma Co., Ltd. (Shanghai, China) according to the manufacturer’s instruction. Briefly, 15 μL of the NC (20 pmol/μL), siPERK (20 pmol/μL), and Lipofectamine™ 2000 (GenePharma Co., Ltd, Shanghai, China) was added to 500 μL of DMEM/F12 medium in a 1.5 mL centrifuge tube. After incubating for 5 min, the Lipofectamine™ 2000 was added to NC or siPERK, mixed thoroughly, and left for 20 min. The culture medium was removed from the cell culture dish, and the cells were washed 3 times with PBS buffer. Then, 4 mL of DMEM/F12 medium along with the prepared complexes were added to each dish. After 6 h of incubation at 37 °C, 550 μL of FBS was added to each dish.

### 2.9. Liquid Chromatography–Tandem Mass Spectrometry (LC-MS/MS) Analysis

The resuspended Granulosa cell pellet precipitate was digested by trypsin in 1 mL of 80% methanol (cooled in an ice bath), treated with ultrasonic waves using a Misonix XL-2000 probe sonicator, and centrifuged at a speed of 28,672× *g* for 10 min at 4 °C. The supernatant was transferred to a CentriVap refrigerated centrifugal concentrator to dry, and then reconstituted with 50 μL ddH_2_O to prepare a series of 20-fold dilutions for the parallel quantitation of itaconic acid using a Waters Acquity H-ClassUPLC System coupled to a Xevo TQ-XS Triple LC-MS/MS System.

### 2.10. Ellman Assay

All experimental procedures were performed using the AChE detection kit (BOXBIO, Beijing, China) according to the manufacturer’s instructions.

### 2.11. Statistical Analysis

Statistical analysis was performed using GraphPad Prism 8 (GraphPad Software, Inc., San Diego, CA, USA). The difference between the groups were analyzed using Student’s *t*-test or one-way analysis of variance (ANOVA) and Tukey’s post hoc test. The data are expressed as mean ± standard deviation. Statistical significance was established when *p* < 0.05. Each experiment was repeated 3 times.

## 3. Results

### 3.1. Effect of Tm on Bovine Granulosa Cells

**Figure 1:**
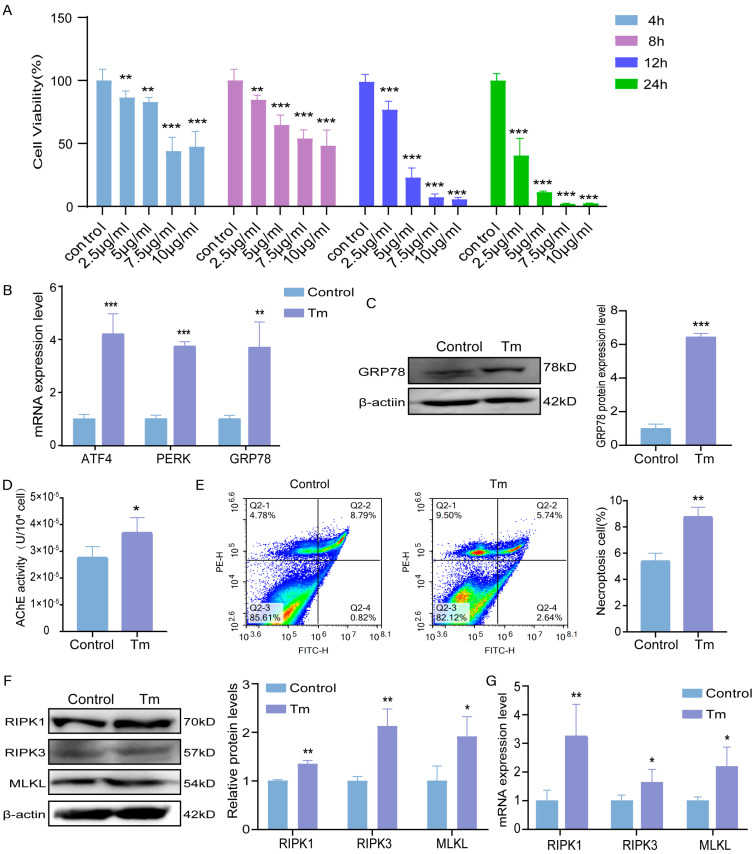
Effect of Tm on bovine granulosa cells. (**A**) Cell survival rate of bovine ovarian granulosa cells treated with Tm was evaluated using CCK8 assay kit. (**B**) mRNA levels of endoplasmic reticulum-related indexes after Tm treatment. (**C**) Protein levels of GRP78 after Tm treatment and statistical analysis of protein gray values. (**D**) Amount of AChE in bovine ovarian granulosa cells. (**E**) Number of necrotic cells in fourth quadrant of bovine ovarian granulosa cells was observed using flow cytometry. (**F**) Protein levels of necroptosis-related indexes after Tm treatment and statistical analysis of protein gray values. (**G**) mRNA levels of necroptosis-related indexes after Tm treatment. * *p* < 0.05; ** *p* < 0.01; *** *p* < 0.001.

In order to elucidate the role of Tm on the bovine granulosa cells, serial dilutions of Tm (2.5, 5, 7.5, and 10 μg/mL) were used at different time periods (4, 8, 12, and 24 h). The effect was evaluated by checking cell viability. As seen in Figure 1A, Tm significantly decreased bovine granulosa cell viability in dose- and time-dependent manners. We went further by studying the molecular mechanism by which Tm decreased granulosa cell viability. We checked the ERS marker PERK, ATF4, and GRP78 mRNA levels and found that 5 μg/mL Tm treatment for 8 h significantly increased the expression of the ERS markers (Figure 1B). We also found that the protein level of GRP78 was up-regulated considerably in the Tm-treated cells (Figure 1C). We also investigated the potential association between AChE and ERS-induced necroptosis after Tm treatment at 5 μg/mL for 8 h. The results showed a more significant increase in the activity of AChE in the Tm-treated group compared to that of the control group, indicating that ERS activated AChE (Figure 1D). Interestingly, flow cytometry showed that the necroptosis cells in the fourth quadrant were significantly up-regulated after Tm treatment, while the apoptotic cells did not significantly change (Figure 1E). Similarly, the necroptosis markers RIPK1, RIPK3, and MLKL were significantly up-regulated at both the translation and transcriptional levels following Tm treatment (Figure 1F,G, respectively).

### 3.2. Protective Effect of Nec-1 Against Tm on Bovine Granulosa Cells

**Figure 2:**
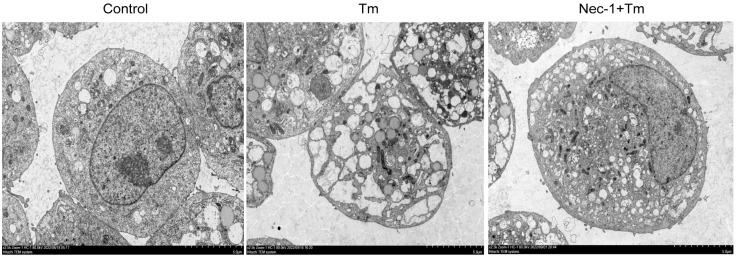
The cell morphology of the granulosa cells in the Tm-treated group and the necroptosis inhibitor Nec-1 pretreatment group were observed by transmission electron microscopy (scale: 5 um).

To further confirm that Tm induced necroptosis in the granulosa cells, the cells were treated with 30 μM necroptosis inhibitor Nec-1 for 12 h, and the cell phenotype was observed by electron microscopy. The results showed marked morphological changes in the Tm-treated granulosa cells, which manifested as the severe vacuolization of the rough endoplasmic reticulum, membrane damage, cytoplasmic translucency, and residual nuclear membrane damage, which not only showed the characteristics of ERS, but also matched the morphological characteristics of necroptosis (Figure 2). Surprisingly, the Nec-1-treated cells tolerated the adverse effect of Tm; as the cytoplasmic membrane of the Nec-1+Tm-treated group was intact, the nucleus was normal, and the endoplasmic reticulum vacuolization was reduced compared to that of Tm (Figure 2). This suggests that Nec-1 treatment alleviates cell death induced by ERS and that ERS can induce necroptosis in bovine granulosa cells.

### 3.3. Protective Effect of HupA Against Tm on Bovine Granulosa Cells

**Figure 3:**
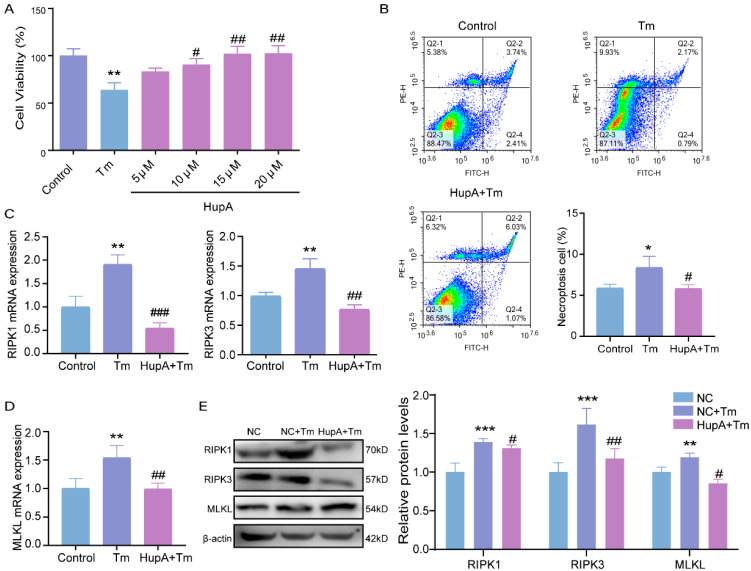
Protective effect of HupA against Tm on bovine granulosa cells. (**A**) Cell viability after HupA pretreatment in bovine ovarian granulosa cells. (**B**) Fourth quadrant necroptosis cell ratio was observed by flow cytometry in bovine ovarian granulosa cells, and necrotic cells were counted. (**C**,**D**) The levels of necroptosis after HupA pretreatment in bovine ovarian granulosa cells. (**E**) Protein levels of necroptosis after HupA pretreatment in bovine ovarian granulosa cells and statistical analysis of protein gray values. * *p* < 0.05; ** *p* < 0.01; *** *p* < 0.001 represents Tm vs. control. # *p* < 0.05; ## *p* < 0.01; ### *p* < 0.001 represents Tm vs. HupA+Tm.

The results demonstrated that HupA treatment at 15 μM and 20 μM significantly improved cell viability, with 20 μM for 24 h selected for the subsequent experiments (Figure 3A). Flow cytometry analysis showed that Tm induced granulosa cell necroptosis, while HupA reduced the rate of cell necroptosis (Figure 3B). Consistent with the flow cytometry results, the protein and gene expression levels of the necroptosis markers RIPK1, RIPK3, and MLKL were significantly down-regulated after HupA treatment (Figure 3C–E).

### 3.4. Knockdown of the IRG1 Exacerbates ERS-Induced Necroptosis

**Figure 4:**
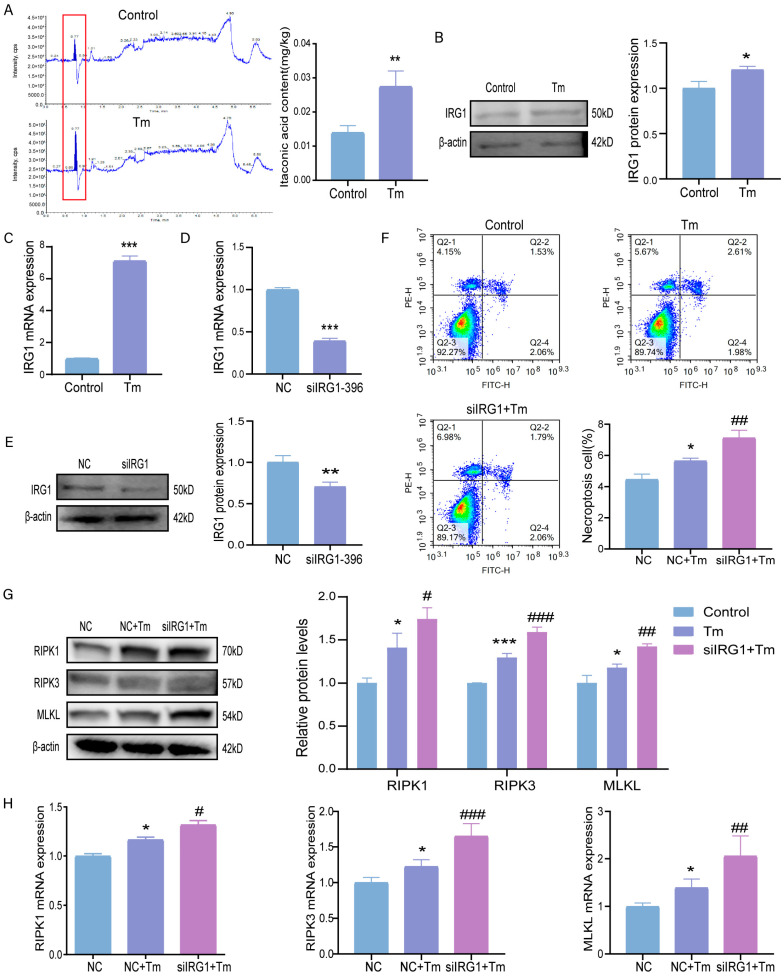
The knockdown of IRG1 exacerbates ERS-induced necroptosis. (**A**) The LC-MS/MS spectra and LC-MS/MS results statistically indicate the level of itaconic acid (red box). (**B**) The protein level and the gray value of IRG1 were determined under ERS. (**C**) The mRNA levels of IRG1 under ERS. (**D**) The mRNA levels of IRG1 after IRG1 interference. (**E**) The protein level and the gray value of IRG1 were statistically determined after IRG1 interference. (**F**) The fourth quadrant necroptosis cell ratio was observed by flow cytometry, and the necrotic cells were counted. (**G**) The protein levels and the gray values of necroptosis were statistically determined after IRG1 interference. (**H**) The mRNA levels of necroptosis after IRG1 interference. * *p* < 0.05; ** *p* < 0.01; *** *p* < 0.001 represents Tm vs. the control. # *p* < 0.05; ## *p* < 0.01; ### *p* < 0.001 represents Tm vs. siIRG1+Tm.

To investigate whether itaconic acid can alleviate ERS-induced necroptosis, we first detected the expression of itaconic acid in the bovine granulosa cells after Tm treatment. The results showed that the expression of itaconic acid was significantly up-regulated under ERS (Figure 4A). We went further by checking the expression of IRG1; the protein and gene expression levels of IRG1 were significantly up-regulated under ERS induced by Tm treatment (Figure 4B,C). We also used siIRG1-396 to knockdown IRG1, and our results showed significant down-regulation in the expression of IRG1 at both the transcriptional and translation levels (Figure 4D,E, respectively). Interestingly, the flow cytometry results showed that IRG1 knockdown exacerbated the ERS-induced necroptosis induced by Tm treatment (Figure 4F). Additionally, the protein and mRNA expression levels of the necroptosis markers RIPK1, RIPK3, and MLKL were significantly up-regulated upon IRG1 knockdown and treatment with Tm (Figure 4G,H).

### 3.5. Effects of Itaconic Acid Supplementation on ERS-Induced Necroptosis in Bovine Granulosa Cells

**Figure 5:**
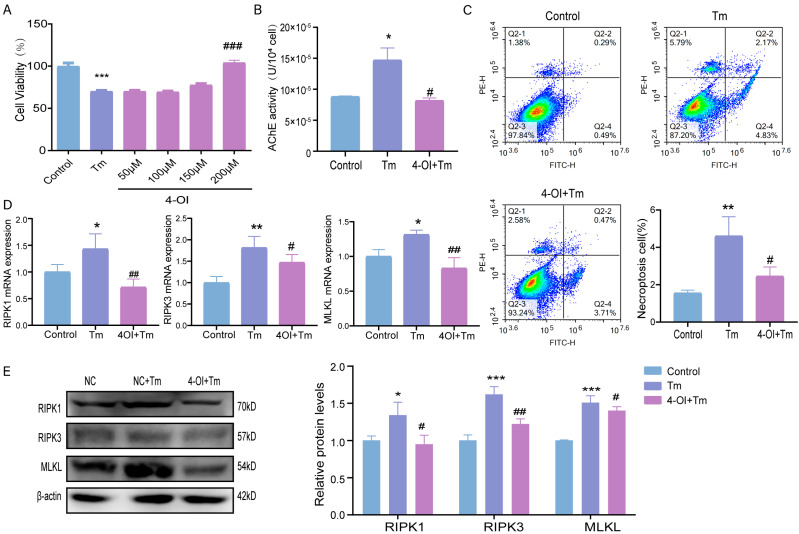
Itaconic acid supplementation alleviates ERS-induced necroptosis in bovine granulosa cells. (**A**) Cell viability after 4-OI pretreatment. (**B**) Amount of AChE after 4-OI pretreatment in bovine ovarian granulosa cells. (**C**) Fourth quadrant necroptosis cell ratio was observed by flow cytometry, and necrotic cells were counted. (**D**) mRNA levels of necroptosis after 4-OI pretreatment. (**E**) Protein levels and gray values of necroptosis after 4-OI pretreatment. * *p* < 0.05; ** *p* < 0.01; *** *p* < 0.001 represents Tm vs. control. # *p* < 0.05; ## *p* < 0.01; ### *p* < 0.001 represents Tm vs. 4-OI+Tm.

To begin, the supplementation of 200 μM of itaconic acid derivative 4-OI more significantly increased cell survival after 12 h compared to that of the Tm group (Figure 5A). Moreover, intracellular AChE expression was significantly down-regulated after the addition of 4-OI, suggesting that itaconic acid may have alleviated the necroptosis induced by Tm treatment (Figure 5B). Similarly, the flow cytometry results showed that 4-OI reduced the rate of ERS-induced cell necroptosis (Figure 5C). The mRNA and protein expression levels of the necroptosis-related indicators RIPK1, RIPK3, and MLKL were significantly reduced after the supplementation of 4-OI, which is consistent with the flow cytometry results (Figure 5D,E).

### 3.6. ERS Activates the PERK-ATF4 Pathway and Stimulates IRG1-AChE to Induce Necroptosis in Bovine Granulosa Cells

**Figure 6:**
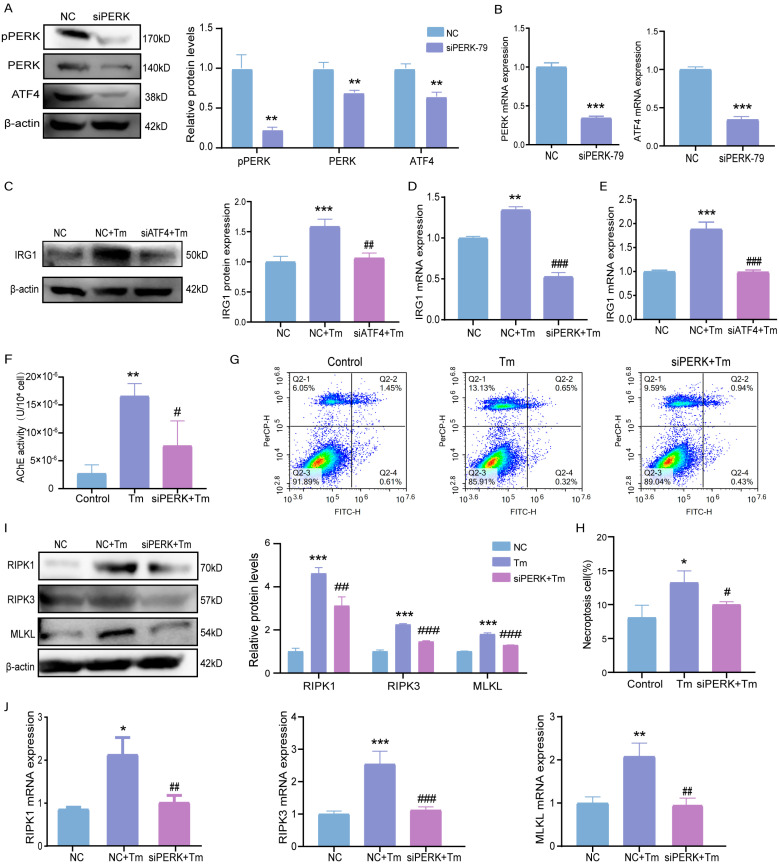
ERS activates the PERK-ATF4 pathway and stimulates IRG1-AChE to induce necroptosis in the bovine granulosa cells. (**A**) The protein level and the gray value of the PERK pathway after interfering with PERK. (**B**) The levels of genes of the PERK pathway after interfering with PERK. (**C**) The protein level and the gray value of IRG1 after interfering with ATF4. (**D**,**E**) The mRNA level of IRG1 after interfering with PERK or ATF4. (**F**) The amount of AChE in bovine ovarian granulosa cells after PERK interference. (**G**,**H**) The fourth quadrant necroptosis cell ratio was observed by flow cytometry. (**I**) The protein level and the gray value of necroptosis were statistically determined after PERK interference. (**J**) The mRNA levels of necroptosis after PERK interference. * *p* < 0.05; ** *p* < 0.01; *** *p* < 0.001 represents Tm vs. the control. # *p* < 0.05; ## *p* < 0.01; ### *p* < 0.001 represents Tm vs. siX+Tm (X = PERK/ATF4).

After PERK knockdown, the expression levels of the PERK and ATF4 genes and proteins were detected. The results showed that the levels of PERK, p-PERK, and ATF4 genes and proteins in the siPERK-treated group were significantly reduced (Figure 6A,B). Additionally, the IRG1 gene and protein expression levels were reduced considerably after PERK or ATF4 knockdown (Figure 6C–E). Also, the AChE level was significantly reduced after PERK knockdown (Figure 6F). Flow cytometry analysis showed a more significant reduction in necroptotic cells after PERK knockdown compared to that of the Tm group (Figure 6G,H). Similarly, the siPERK+Tm group had significantly lower protein and gene expression levels of the necroptosis-related indicators RIPK1, RIPK3, and MLKL (Figure 6I,J).

## 4. Discussion

The rates of oocyte growth and granulosa cell proliferation increase gradually after follicular growth reaches the secondary follicular phase, which can lead to hypoxia [24]. This local condition may lead to endoplasmic reticulum dysfunction, accumulating unfolded and misfolded proteins, which induce ERS and the UPR [25]. Under endocrine and paracrine control, one follicle is selected in a cohort of follicles growing simultaneously, and the other follicles enter follicular atresia [26], which may be induced, in part, by ERS [27]. This study used Tm as an ERS inducer to establish an ERS model and investigate the molecular mechanism of ERS-induced necroptosis in bovine granulosa cells.

Tm mainly triggers ERS by inhibiting the protein glycosylation pathways to initiate the UPR. IRE1 α, PERK, and ATF6, which bind to GRP78, are UPR sensors in the endoplasmic reticulum cavity used to the monitor protein levels of misfolded proteins [28]. The abnormally accumulated misfolded proteins in the endoplasmic reticulum lumen compete with GRP78 for binding [29]. The binding of GRP78 to the lumen domains of IRE1 α, PERK, and ATF6 is inhibited, and GRP78 is released, which, in turn, promotes the activation of these UPR sensors [30,31]. Therefore, we focused on the protein expression of GRP78 and reported that Tm treatment can lead to the activation of the UPR and successfully simulate ERS in granulosa cells. After Tm treatment, we found that the genes and proteins, which are the key indicators of necroptosis under ERS, were significantly up-regulated, suggesting that ERS may cause follicular atresia by inducing necroptosis in bovine granulosa cells. This is consistent with the results of intestinal, auditory, and human ovarian granulosa cells [15,32,33].

The necroptosis inhibitor Nec-1 treatment was used to inhibit necroptosis to rescue cell mortality and alleviate cell phenotypic changes. Nec-1 did not alleviate the cell phenotype of crude endoplasmic reticulum vacuolization, but alleviated the cell phenotype caused by necroptosis. This fully proved that ERS participates in bovine follicular atresia by inducing the necroptosis of granulosa cells.

Under ERS conditions, the PERK pathway is activated and induces necroptosis; this pathway triggers an increase the level of pro-apoptotic C/EBP homologous protein (CHOP) and promotes the propagation of ROS signals between the endoplasmic reticulum and mitochondria through its tethered function, thereby promoting apoptosis [34,35]. ATF4 also mediates the necroptosis of rhabdomyosarcoma induced by starvation [36], which is a potent ERS inducer. Based on our study results, we verified that the gene and protein expressions of the key indicators of necroptosis were significantly reduced after interfering with PERK under ERS, demonstrating that the PERK-ATF4 pathway mediates ERS-induced necroptosis in bovine granulosa cells.

Endogenous small molecules are the metabolic regulators of cell function. Itaconate is a key molecule that accumulates in cells when the Krebs cycle is disrupted. Itaconic acid is synthesized by immune response gene 1 (IRG1) in the mitochondrial matrix [23]. Some studies have shown that in addition to its significant anti-inflammatory and antioxidant effects [37], itaconic acid can also alleviate ERS through the Nrf2 pathway [38]. In the ovaries, itaconic acid can be present in follicular fluid [39], and it can improve ovarian dysfunction caused by granulosa cell inflammation in dairy cows [40]. In this study, it was found that IRG1 is involved in ERS-induced necroptosis and is regulated by the PERK pathway in bovine granulosa cells, while the exogenous addition of itaconic acid and its derivatives can alleviate ERS-induced necroptosis.

AChE can be expressed in human granulosa cells and is closely related to cell death [2]. The inhibition of AChE activity can enhance follicular development and fertility in rats [41]. This matches with our study that demonstrated the over-expression of AChE activity in bovine granulosa cells under ERS. In addition, AChE inhibitors are mainly classified into two categories, those that induce or do not induce ERS, and the latter inhibitor leads to the retention and accumulation of misfolded AChE in the endoplasmic reticulum [42]. To avoid the interference of AChE inhibitors on the ERS model, the cells were treated with HupA, a non-inducing ERS AChE inhibitor, combined with Tm to detect cell damage, and it was found that AChE participates in the necroptosis of bovine granulosa cells induced by ERS and is regulated by the PERK-IRG1 pathway.

## 5. Conclusions

**Figure 7:**
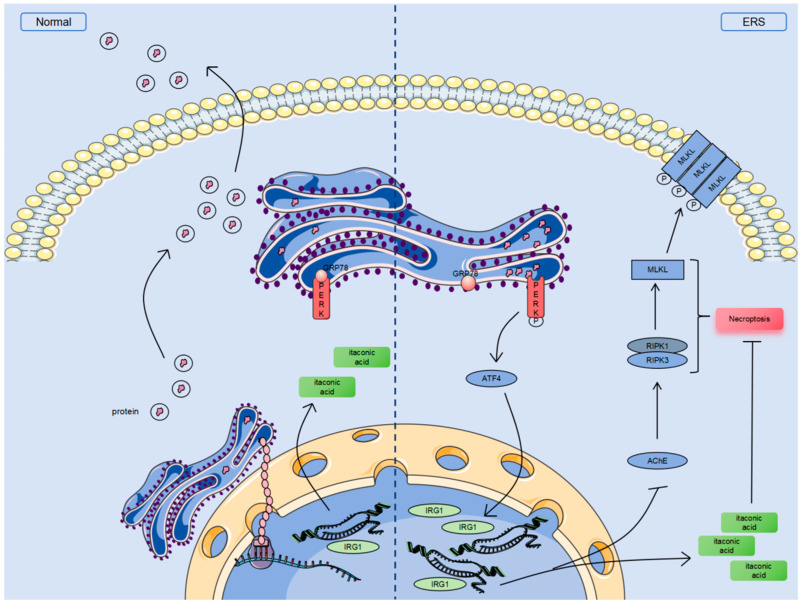
Schematic diagram of mechanism of action of IRG1/itaconic acid/AChE in ERS-induced necroptosis of bovine ovarian granulosa cells.

In summary, we reported that ERS activates the PERK-ATF4 pathway, promotes the expression of IRG1, and exacerbates the necroptosis caused by acetylcholinesterase AChE in bovine granulosa cells. The exogenous supplementation of the itaconic acid derivative 4-OI can alleviate ERS damage to granulosa cells (Figure 7). These findings shed the light on novel innovative approaches for improving animal reproductive efficiency.

ERS exacerbates the necroptosis caused by acetylcholinesterase AChE by activating the PERK-ATF4 pathway. The activation of the PERK-ATF4 pathway promotes the expression of the IRG1 gene and generates metabolic small-molecule itaconic acid, which can alleviate necroptosis.

## Data Availability

The data presented in this study are available on request from the corresponding author upon reasonable request.

## Supplementary Materials

The following supporting information can be downloaded at: https://www.mdpi.com/article/10.3390/cells14060419/s1, Table S1: Primer sequences used in qRT-PCR analysis; Table S2: List of antibodies for Western Blot analysis; Table S3: Sequence of transfection fragments.

## Abbreviations

ERS	endoplasmic reticulum stress
Tm	tunicamycin
AChE	acetylcholinesterase
UPR	unfolded protein response
IRG1	immune-responsive gene 1
4-OI	4-Octyl itaconate
CHOP	C/EBP homologous protein
