# Supplementary material for: Ameliorative Effect of Itaconic Acid/IRG1 Against Endoplasmic Reticulum Stress-Induced Necroptosis in Granulosa Cells via PERK-ATF4-AChE Pathway in Bovine
Source: Cells. 2025 Mar 12;14(6):419. doi: 10.3390/cells14060419 (PMC11940906; doi:10.3390/cells14060419)
Supplement: Supplementary file 1 [file cells-14-00419-s001.zip › cells-3492808-supplementary.pdf]

Table S1 Primer sequences used in qRT-PCR analysis

| Gene | Primer sequences (5'→3') | Tm（℃） |
|---|---|---|
| GRP78 | F: CGTGCGTTTGAGAGCTCAGT | 58 |
| | R: TAGGGCTTCGCAGGAAAACC | |
| ATF4 | F: ACATCATGGGTTCTCCTGCG | 56 |
| | R: AAGCATCCTCCTTGCTGTTGT | |
| ATF6 | F: AGTACGATGAAAAATATTGCTGTGT | 56 |
| | R: CCGCTTCAGGGAACCATTCT | |
| PERK | F：CCAGCAAAGAGGAGCCCAGAATG | 60 |
| | R: AAGTGGTTGGTCTTGACGGAGAAAC | |
| RIPK1 | F；GCGAATCTCTCGGGTTGTGT | 57 |
| | R：TTCAGGATCACCAGTCCGTG | |
| RIPK3 | F: GTCCACATTTCAGGGAGGCT | 57 |
| | R: GAAGGATCCCAGAGTCTGTCT | |
| MLKL | F: GGGAGCAGCACTTCTCTGTTA | 57 |
| | R: GGGGCTGCTAAGTCACAATG | |
| IRG1 | F: ATCTTCACAGAGCGTTCGGATACC | 61 |
| | R: TTTCTACAGCGTGGCAGGACAG | |
| β-actin | F: TTGATCTTCATTGTGCTGGGTG | / |
| | R: CTTCCTGGGCATGGAATCCT | |

Table S2 List of antibodies for Western Blot analysis

| Antibodies | Company | Catalogue code | Dilution |
|---|---|---|---|
| Anti-GRP78 | Proteintech | 11587-1-AP | 1:1000 |
| Anti-RIPK1 | Proteintech | 17519-1-AP | 1:1000 |
| Anti-RIPK3 | ABclonal | A5431 | 1:1000 |
| Anti-MLKL | ABclonal | A13450 | 1:1000 |
| Anti-PERK | Cell Signaling | D11A8 | 1:1000 |
| Anti-pPERK | Affinity | DF7576 | 1:1000 |
| Anti-ATF4 | Bioss | bs-1531R | 1:1000 |
| Anti-IRG1 | Proteintech | 28436-1-AP | 1:1000 |
| Anti-β-actin | Proteintech | 60008-1-1g | 1:60000 |

Table S3 Sequence of transfection fragments

| serial | sense（3'→5'） | antisense（5'→3'） |
|---|---|---|
| PERK-Bos-1679 | GCAGAUCACGGUCAGAUUUTT | AAAUCUGACCGUGAUCUGCTT |
| ATF4-Bos-427 | CCCAGAAGGUUUACCAACAATT | UUGUUGGUAAACCUUCUGGTT |
| IRG1-Bos-396 | GGCUUUCAAUGUUGGUAUUTT | AAUACCAACAUUGAAAGCCTT |
| NC | UUCUCCGAACGUGUCACGUTT | ACGUGACACGUUCGGAGAATT |
